# Epizootic Hemorrhagic Disease in Brocket Deer, Brazil

**DOI:** 10.3201/eid1902.120551

**Published:** 2013-02

**Authors:** Cíntia Maria Favero, Ana Carolina Diniz Matos, Fábia Souza Campos, Marcus Vinícius Cândido, Érica Azevedo Costa, Marcos Bryan Heinemann, Edel Figueiredo Barbosa-Stancioli, Zélia Inês Portela Lobato

**Affiliations:** Author affiliations: Universidade Federal de Minas Gerais, Belo Horizonte, Brazil (C.M. Favero, A.C.D. Matos, F.S. Campos, É.A. Costa, M.B. Heinemann, E.F. Barbosa-Stancioli, Z.I.P. Lobato);; Universidade de São Paulo, São Paulo, Brazil (C.M. Favero);; Pomedore Zoo, Pomedore, Brazil (M.V. Cândido)

**Keywords:** epizootic hemorrhagic disease, orbivirus, brocket deer, Brazil, epizootic hemorrhagic disease virus, viruses, deer, *Mazama gouazoubira*

**To the Editor:** In February 2008, a 2-year-old female gray brocket deer (*Mazama gouazoubira*) at Pomerode Zoo in Santa Catarina, Brazil, exhibited sublingual swelling, drooling, lethargy, prostration, glossitis, slight cyanosis, and blood on the perineum. The animal showed progressive anorexia, ataxia, dyspnea, marked cyanosis, hypothermia (34.5°C), and severe anemia (hematocrit 4.6%). The deer died 5 days after the first clinical signs. Necropsy indicated that the gray brocket deer had pulmonary congestion and edema, mild renal congestion, and renal focal necrosis. Some intestinal vessels along the serosa presented with hyalinization of the walls with mild leukocytic infiltrate of neutrophils featuring vasculitis.

Seventeen days after this first case, a 1-year-old male pygmy brocket deer (*Mazama nana*) from the same zoo suddenly died. Findings on necropsy were cyanosis and petechiae in the oral mucosa, tongue, and gastrointestinal mucosa. There was bloody intestinal content, petechiae in the mucosa of the urinary bladder, and also petechiae and ecchymoses in the pericardium and epicardium. The spleen was contracted; lymph nodes and kidney medullae were hemorrhagic. The lungs showed congestion and petechiae, and the airways had a frothy fluid content. Histopathologic lesions included mild diffuse congestion in the pygmy brocket deer’s kidneys and extensive subendocardial hemorrhage. 

To identify the suspected disease agent (members of the species *Bluetongue virus* or *Epizootic hemorrhagic disease virus*), we performed virus isolation and reverse transcription PCR on tissues (heart, liver, lung, and bowel) from the pygmy brocket deer. (Virus isolation was not carried out on specimens from the gray brocket deer because brain tissue samples were inadequate and results of PCR were negative for epizootic hemorrhagic disease virus [EHDV] or bluetongue virus [BTV].) Infection with EHDV and BTV was first diagnosed by virus isolation. Pooled specimens from spleen-liver and heart tissue were inoculated in embryonated chicken eggs and thereafter in BHK-21 cells (*1*). Chicken embryos inoculated with tissues of the pygmy brocket deer died from 1 to 6 days after inoculation. All embryos showed swelling and hemorrhage throughout the skin and extensive areas of hemorrhage in the brain and heart. Cytopathic effects started 48 hours postinoculation in the BHK-21 monolayer. An indirect immunoperoxidase test, using anti-BTV/EHDV polyclonal antiserum of porcine origin (VMRD Inc., Pullman, WA, USA) and direct fluorescence assay using an anti-BTV monoclonal antiserum fluorescein conjugate (VMRD) were performed for virus identification (*1*). The isolates were then identified as EHDV.

To confirm the EHDV serogroup, we performed reverse transcription PCR. A fragment of ≈260 bp, which encodes the partial NS3 gene of EHDV, was detected from pooled tissue and the BHK-21 monolayer with cytopathic effect (*2*). No amplification was obtained in the S10 gene PCR for BTV (*3*). An amplified fragment was sequenced and identified as strain LDVA (GenBank accession no. GU014478). The phylogenetic relationship was assessed by using the neighbor-joining Mega.5 (*4*) method with the BTV sequence as an outgroup. The phylogenetic tree shows that the partial sequence of the S10 gene segregates EHDV serogroup into 2 clusters, with LDVA (GU014478) grouping together with North American EHDV samples (Figure).

**Figure:**
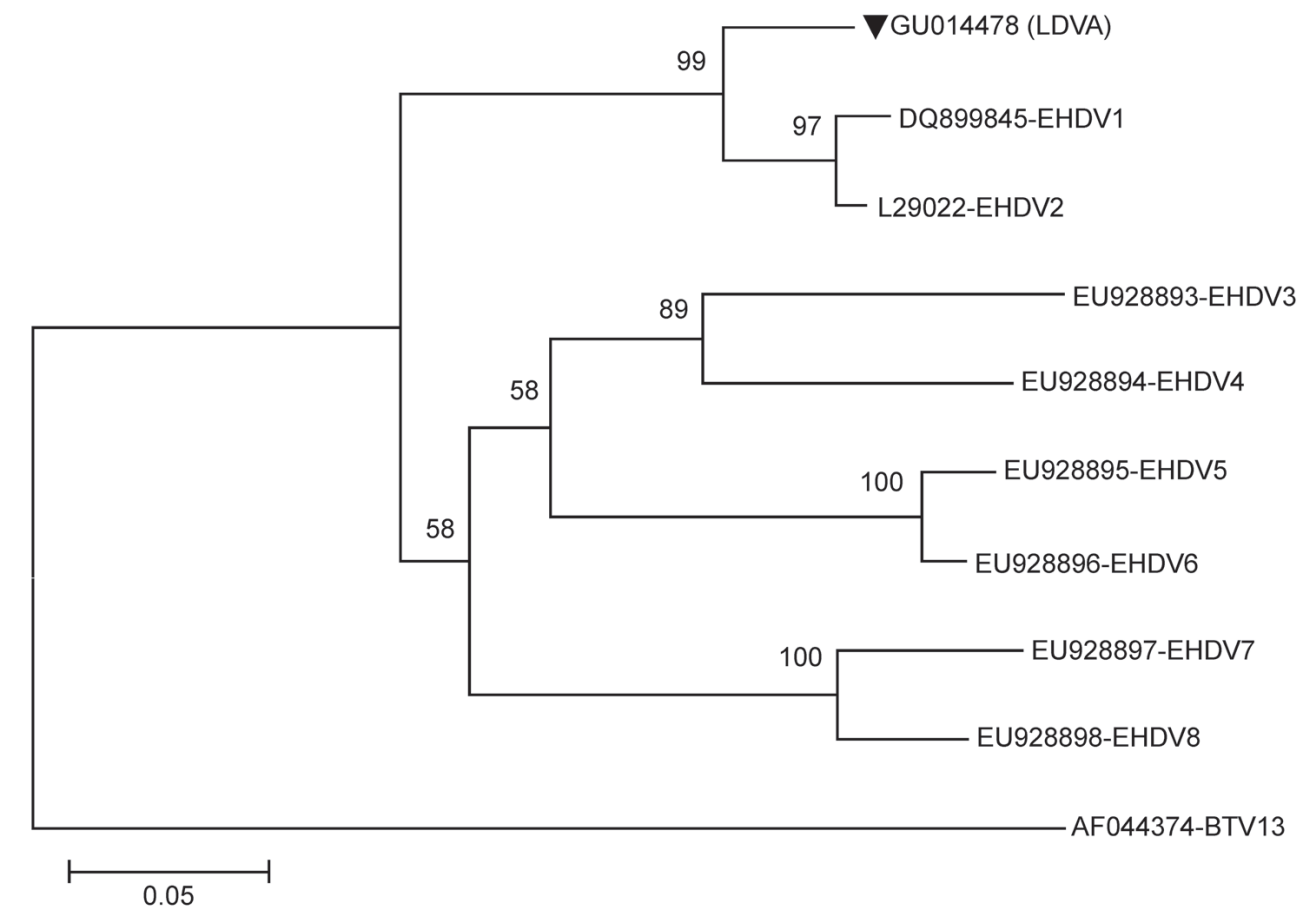
Phylogenetic tree based on a 244-bp epizootic hemorrhagic disease virus (EHDV) NS3 gene. GU014478 (GenBank accession no.; LDVA) strain (triangle) isolated from pygmy brocket deer in the present study (southern Brazil, 2008), New Jersey strain DQ899845 (EHDV1), Alberta strain L29022 (EHDV2), Nigeria strain EU928893 (EHDV3), Nigeria strain EU928894 (EHDV4), Australia strain EU928895 (EHDV5), Australia strain EU928896 (EHDV6), Australia strain EU928897 (EHDV7), Australia strain EU928898 (EHDV8), and bluetongue virus (BTV) 13 outgroup USA strain AF044374. Scale bar indicates nucleotide substitutions per site.

EHDV and BTV (*Reoviridae: Orbivirus*) are involved in outbreaks of hemorrhagic disease, which occur mainly in late summer and early autumn in various parts of the world, probably because of the increase in the *Culicoides* population, the biologic vectors of these viruses. Clinical signs of EHDV and BTV infections are indistinguishable in deer and are characterized by severe depression, respiratory distress, anorexia, and blood-tinged oral and nasal discharge. Gross lesions are mainly edema and hemorrhage caused by vascular injury (*5*).

Clinical reports of EHDV infection have occurred in Australia, Asia, Africa, and North America (*6*). In Brazil, serologic research in marsh deer (*Blastocerus dichotomus)* populations in São Paulo and Mato Grosso do Sul revealed that 74% of animals were seropositive for EHDV, 88% were seropositive for BTV, and 60% were seropositive for both viruses (*7*). Deaths of 6 marsh deer that showed clinical signs and gross and microscopic lesions resembling EHDV/BTV infection were described, but not confirmed, in São Paulo (*8*). Although EHD is endemic in the country (*7*,*8*), in the southern region of the Brazil, no serologic reports of EHDV have been made, and the serologic prevalence of BTV in domestic ruminants is low, ≈0.2% (*9*). Climatic factors can contribute to this distribution because temperatures in southern Brazil are low in autumn and winter and thus unfavorable to the development and survival of competent species of *Culicoides* biting midges. This low serologic prevalence probably produces an area of enzootic instability in which epidemic cases occur that are associated with the presence of infected vectors and susceptible animals (*9*).

In summary, we describe the isolation of EHDV that is involved in hemorrhagic disease affecting pygmy brocket deer in Brazil. The hazard that this virus infection could pose to the local and national Cervidae populations remains unknown, but it is noteworthy that *M. nana* deer are the most threatened species in Brazil (*10*). For better control and prevention measures to be developed, research is needed to characterize Brazilian EHDV isolates and variations in resistance at the species and subspecies level. 
